# Phenotypic modulation of pentylenetetrazole-induced convulsive behaviors in *C. elegans* carrying a mutation associated with Alzheimer’s disease

**DOI:** 10.17912/micropub.biology.000295

**Published:** 2020-08-20

**Authors:** Madeline A. Vaji, Guy A. Caldwell, Kim A. Caldwell

**Affiliations:** 1 Department of Biological Sciences, The University of Alabama, Tuscaloosa, AL 35487-0344

**Figure 1. Comparing convulsions induced by PTZ using both liquid-based and plate-based assays with the indicated concentrations of PTZ:**
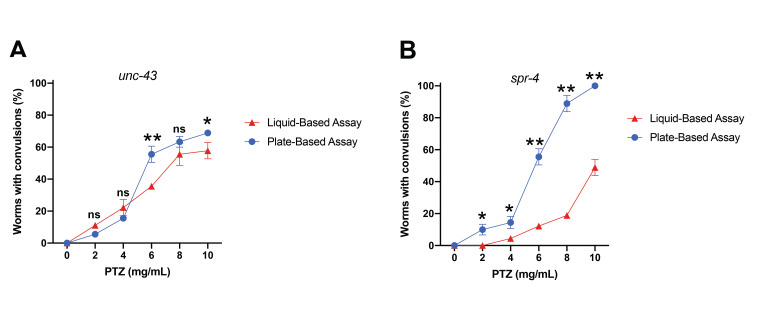
(A) Populations of control, *unc-43(n498 n1186),* mutant animals exposed to PTZ on plates (blue line) and liquid (red) displayed similar rates of convulsions with only a few concentrations displaying differences in convulsion rates between the assays. (B) Populations of *spr-4(by105)* mutant animals exposed to PTZ on plates consistently exhibited greater rates of convulsions compared to animals in the liquid-based assay; ns = not significant; *p<0.005; **p<0.0001 Two-Way ANOVA with a Sidak’s post-hoc analysis.

## Description

The restrictive element-1 silencing transcription factor (REST)/neuron-restrictive silencing factor (NRSF) is a transcriptional repressor which actively suppresses genes involved in a wide array of neuronal processes such as global ischemia, neurodegenerative conditions and seizure disorders (Calderone *et al.*, 2003; Lu *et al.*, 2014; McClelland *et al.*, 2011). In *C. elegans*, *spr-4* is the homolog of REST; a mutation in *spr-4(by105)* enhanced amyloid-beta-induced neurotoxicity of glutamatergic neurons in a worm Alzheimer’s disease model (Lu *et al.*, 2014). Previously published research in mammals has reported that REST contributes to the onset of epilepsy via repression of specific genes (McClelland *et al.*, 2011). Additionally, there is a 2- to 6-fold increase in the prevalence and incidence of seizures in Alzheimer’s disease patients (Nicastro *et al.*, 2016). Taken together, we hypothesized there might be a REST-dependent convulsion phenotype in *spr-4(by105)* mutants. We previously developed a protocol for studying epileptic-like convulsions in *C. elegans* that involved treating worms with the GABA antagonist, pentylenetetrazole (PTZ) on agar plates (Locke *et al.*, 2008). Here, we tested *spr-4(by105)* worms for convulsions using our previously developed agar plate assay and a newly developed liquid-based assay from another lab (Wong *et al.*, 2018), both of which used PTZ to induce convulsions. As a negative control, we examined N2 wild-type animals. It should be noted that these wild-type animals did not exhibit a single convulsion at any concentration (2-10 mg/mL PTZ) with either the liquid or plate-based assay. For both assays, we used the *unc-43(n498 n1186)* worms as a positive convulsion control (Fig. 1A) as these animals are responsive to PTZ on agar plates (Williams *et al.*, 2004) and in liquid containing PTZ (Wong *et al.*, 2018). Notably, the behavioral response of the *unc-43* animals was similar in both assay types. The *unc-43* animals had more active phenotypes at higher concentrations (6, 8, and 10 mg/mL PTZ) where they displayed a “rubberband”, or clonic convulsion, phenotype. At lower concentrations (4 mg/mL) the animals exhibited either a rubberband or paralysis phenotype. Paralyzed animals still displayed pharyngeal activity and completely recovered normal movement within 20-30 minutes following removal from PTZ. These control animals informed our experimental conditions. We wanted to determine if the convulsion response in *spr-4* mutant worms would be similar between the assays or preferentially modulated. Notably, populations of *spr-4* mutant animals displayed significantly different convulsion levels at every concentration of PTZ in response to these alternate assay conditions (Fig. 1B). In all cases, the population of animals responsive to PTZ was significantly lower using the liquid-based assay in comparison to the plate-based assay. With both assays, however, the *spr-4* worms did display similar types of phenotypic convulsions at comparable concentrations. Specifically, at higher concentrations of PTZ (8 and 10 mg/mL) *spr-4* worms exhibited “tonic-clonic”, or head-bobbing, convulsions where the anterior region of the animal had a repetitive movement while the posterior was paralyzed. Conversely, at lower concentrations of PTZ (2 and 4 mg/mL), *spr-4* animals displayed primarily full body paralysis. We do not know why *spr-4* animals displayed such stark differences in convulsion levels following exposure to PTZ from these two assay types when such distinct and contrasting variability was not displayed by the *unc-43* worms. However, it is known that environmental circumstances can induce convulsions in humans. In this regard, we suggest that in our worm assays, mechanosensory or osmotic stress differences might modulate the PTZ-induced epileptic-like convulsive behavior of certain *C. elegans* mutants in an assay and concentration-dependent manner.

## Methods

**Plate-based assay**. 60 mm NGM agar plates were prepared 48 hours before the assay and stored in a 20^o^C incubator. A PTZ stock of 0.5 g/mL (Sigma) in ddH_2_O was prepared fresh on the day of analysis. Appropriate concentrations of PTZ (2-10 mg/mL) were applied to the tops of the NGM plates with a cell spreader/hockey stick. PTZ was dissolved in water; thus, solvent-only (0 mg/mL) plates were also prepared as controls. The plates were dried for 60 minutes with the lids ajar in a sterile hood. The PTZ plates were then seeded with *E. coli* OP50, which was dried for 30 minutes, in a sterile hood, with the lids ajar, before use. Three plates per concentration/strain were prepared, as each mutant was examined in triplicate. For each mutant, 30 animals were then transferred/plate and examined for convulsion activity (Locke *et al.*, 2008). As worms exhibited convulsion phenotypes, they were removed from the plate and the type of convulsion phenotype and time of the convulsion was noted.

**Liquid-based assay**. Liquid for *C. elegans* immersion consisted of Dent’s Ringer solution (DRS). Thirty day 3, young adult worms per experimental condition were placed in 50 μL liquid droplets of different concentrations of PTZ (2-10 mg/mL) dissolved in DRS containing 0.1% bovine serum albumin placed on plastic Petri dish lids. The worms were incubated in this solution for 30 minutes. Following the incubation period, the worms were transferred from the droplet with an eyelash pick and placed on to an NGM plate seeded with 25 μL of *E. coli* OP50. Worms were then observed for convulsive phenotypes for 1 minute (Wong *et al.*, 2018). As behavioral phenotypes were identified, worms were removed. Each strain was examined in triplicate, with 30 animals examined/replicate, for a total of 90 animals.

**Statistics.** For either assay, statistical analysis was performed with a Two-Way ANOVA with a Sidak’s *post-hoc* analysis (GraphPad).

## Reagents

*C. elegans* strain MT2605, *unc-43(n498 n1186)*

*C. elegans* strain LA95, *spr-4(by105)*

*E. coli* strain OP50 (saturated culture, previously grown in LB and stored at 4°C)

Pentylenetetrazole (Sigma)

Cell spreader/hockey stick (Thomas Scientific, 7012Q52)

Dent’s Ringer solution (DRS), 140 mM NaCl, 1 mM MgCl_2_, 3 mM CaCl_2_, 6 mM KCl, 10 mM HEPES, pH 7.4.

## References

[R1] Calderone A, Jover T, Noh KM, Tanaka H, Yokota H, Lin Y, Grooms SY, Regis R, Bennett MV, Zukin RS (2003). Ischemic insults derepress the gene silencer REST in neurons destined to die.. J Neurosci.

[R2] Locke C, Berry K, Kautu B, Lee K, Caldwell K, Caldwell G (2008). Paradigms for pharmacological characterization of C. elegans synaptic transmission mutants.. J Vis Exp.

[R3] Lu T, Aron L, Zullo J, Pan Y, Kim H, Chen Y, Yang TH, Kim HM, Drake D, Liu XS, Bennett DA, Colaiácovo MP, Yankner BA (2016). Addendum: REST and stress resistance in ageing and Alzheimer's disease.. Nature.

[R4] McClelland S, Flynn C, Dubé C, Richichi C, Zha Q, Ghestem A, Esclapez M, Bernard C, Baram TZ (2011). Neuron-restrictive silencer factor-mediated hyperpolarization-activated cyclic nucleotide gated channelopathy in experimental temporal lobe epilepsy.. Ann Neurol.

[R5] Nicastro N, Assal F, Seeck M (2016). From here to epilepsy: the risk of seizure in patients with Alzheimer's disease.. Epileptic Disord.

[R6] Williams SN, Locke CJ, Braden AL, Caldwell KA, Caldwell GA (2004). Epileptic-like convulsions associated with LIS-1 in the cytoskeletal control of neurotransmitter signaling in Caenorhabditis elegans.. Hum Mol Genet.

[R7] Wong SQ, Jones A, Dodd S, Grimes D, Barclay JW, Marson AG, Cunliffe VT, Burgoyne RD, Sills GJ, Morgan A (2018). A Caenorhabditis elegans assay of seizure-like activity optimised for identifying antiepileptic drugs and their mechanisms of action.. J Neurosci Methods.

